# Acute effects of caffeine withdrawal on headache among regular caffeinated coffee drinkers

**DOI:** 10.1038/s41598-026-54049-3

**Published:** 2026-05-22

**Authors:** Lora Randa, Catherine Lee, David G. Rosenthal, S. Andrew Josephson, Emily Wilson, Jeffrey E. Olgin, Gregory M. Marcus

**Affiliations:** 1 Division of Cardiology, University of California, San Francisco, 505 Parnassus Ave, M1180B, San Francisco, CA 94143 USA; 2 Department of Epidemiology and Biostatistics, University of California, San Francisco, 505 Parnassus Ave, M1180B, San Francisco, CA 94143 USA; 3 Department of Neurology, University of California, San Francisco, 505 Parnassus Ave, M1180B, San Francisco, CA 94143 USA; 4 Swedish Heart & Vascular - Cherry Hill Arrhythmia and Device Clinic, Seattle, WA USA

**Keywords:** Caffeine, Caffeine withdrawal, Headache, Diseases, Health care, Medical research, Neurology, Neuroscience

## Abstract

Headaches are among the most common neurological disorders, often leading to significant disability and reduced quality of life^1,2^. However, much remains unknown about the triggers that precipitate headaches^3^. Coffee is one of the most widely consumed beverages worldwide, and coffee withdrawal is thought to be associated with headache^4,5^. However, research linking caffeine withdrawal to headache in healthy adult populations is largely limited to observational studies prone to confounding or single snapshot assessments prone to recall bias^6–8^. While small, near-term randomized trials have been conducted, these generally are done in artificial study-based settings (rather than among ambulatory individuals) and fail to capture repeated events that best represent real-life circumstances^9,10^. We sought to leverage a prospective, randomized, case-crossover trial to assess acute relationships between caffeine withdrawal and headaches among healthy individuals^11^. Here, we show that among regular caffeinated coffee consumers, caffeine avoidance likely contributes to headaches. When headaches are present, caffeine avoidance was also associated with increased headache severity. These results suggest that headache, commonly experienced by a substantial proportion of the population, may often occur as a consequence of caffeinated coffee withdrawal. Together, these findings may inform behaviors and recommendations regarding coffee consumption and minimizing headache burden on a day-to-day basis.

**Supplementary Information:**

The online version contains supplementary material available at 10.1038/s41598-026-54049-3.

## Introduction

Headaches are highly prevalent neurological disorders that lead to significant disability and reduced quality of life^1,2^. Acute headaches are one of the leading causes of emergency department (ED) visits in the United States, representing a significant healthcare cost burden^3,4^. Independent of such readily measurable effects, headaches that do not necessarily come to clinical attention can substantially reduce quality of life and may reduce work productivity^5–7^. However, much remains unknown about the triggers that precipitate headaches^8^. Better characterizing the acute triggers of headache may uncover readily modifiable strategies to avoid headaches and allow for understanding of mechanisms that themselves may reveal novel targets for headache treatment.

Coffee is among the most widely consumed beverages worldwide^9^. 85% of adults consume caffeine daily in the United States, with coffee being the most common source of caffeine intake^10,11^.

Caffeine withdrawal is thought to be associated with headache^12^. However, research linking caffeine withdrawal to headache in ambulatory adult populations is largely limited to observational studies prone to confounding or single snapshot assessments (cross-sectional studies) prone to recall bias^13–15^. While small, near-term randomized trials have been conducted, these generally are done in artificial study-based settings (rather than among ambulatory individuals) and fail to capture repeated events that best represent real-life circumstances^16,17^. We sought to leverage a prospective, randomized, case-crossover trial to assess acute relationships between caffeine withdrawal and headaches among healthy individuals that regularly consumed caffeinated coffee^18^.

## Results

The baseline characteristics of the research participants stratified by the presence or absence of reporting baseline headaches in response to caffeine withdrawal are shown in Table 1. They were generally demographically representative of the city of San Francisco. At baseline, most participants did not experience caffeine withdrawal headaches. The amount of caffeinated coffee consumed prior to study participation ranged from less than one cup per month to four to five cups daily.

**Table 1:** Participant Characteristics Stratified by Baseline Headaches with Caffeine Abstinence.

Baseline Characteristic	Experiences headaches with 16 + hours of coffee abstinence at baseline		*P*-value
	**Yes** (*n* = 42)	**No** (*n* = 58)	0.11
Age (years)	40 ± 12	39 ± 14	0.81
Female - (%)	22 (52%)	29 (50%)	0.97
Race - (%)			
White	24 (57%)	29 (50%)	0.57
Black	5 (12%)	3 (5%)	0.34
Asian	11 (26%)	23 (40%)	0.24
Pacific Islander	0 (0%)	1 (2%)	0.48
Other	2 (5%)	3 (5%)	0.69
Hispanic Ethnicity – (%)	3 (7%)	5 (9%)	1.0
BMI (kg/m2), median IQR	24, 22–26	24, 22–26	0.81
Hypertension – (%)	3	2	0.50
Diabetes mellitus – (%)	0 (0%)	1 (2%)	0.48
Coronary artery disease – (%)	0 (0%)	0 (0%)	1.0
Usual Coffee Drink Frequency – (%)			
Less than one cup per month	0 (0%)	5 (9%)	0.07
1–3 cups per month	0 (0%)	6 (10%)	0.04
2–4 cups per week	2 (5%)	13 (22%)	0.03
6–7 cups per week	6 (14%)	16 (28%)	0.18
1 cup per day	17 (40%)	11 (19%)	0.03
2–3 cups per day	15 (36%)	6 (10%)	0.0047
4–5 cups per day	2 (5%)	1(2%)	0.78

Participants remained in the study a median of 14 (IQR 13–14) days, and 74% completed all 14 days. The number of participants who completed less than 14 study days and reasons for early withdrawal are listed in Supplementary Table S1. During the study, all 100 participants completed at least one headache questionnaire; headache questionnaires were completed on 89% of participant study days. Participants responded to the headache questionnaire on a median of 13 (IQR 11–14) days (or a median of 93%, IQR 85–100%, days of their study participation).

### Presence or absence of headache: intention-to-treat

**Fig. 1:**
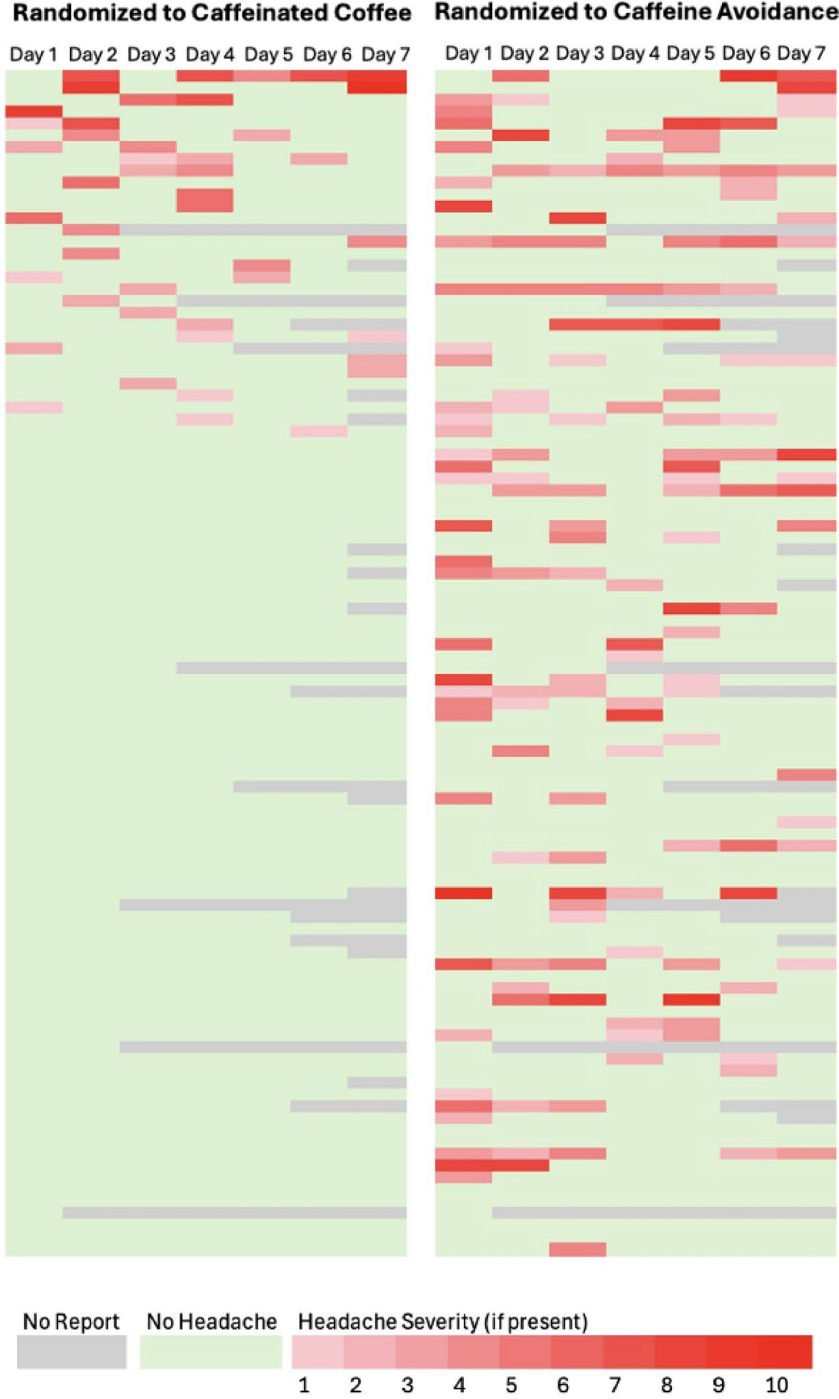
Heatmap of Headache Incidence and Severity by Caffeinated Coffee Randomization Assignment. Each row corresponds to an individual participant, columns represent daily headache observations over the 14-day study period. Only days randomized to consume caffeinated coffee in chronologically order are shown on the left; only days randomized to caffeinated coffee consumption in chronologically order are shown on the right. Color corresponds to self-reported headache severity.

**Fig. 2:**
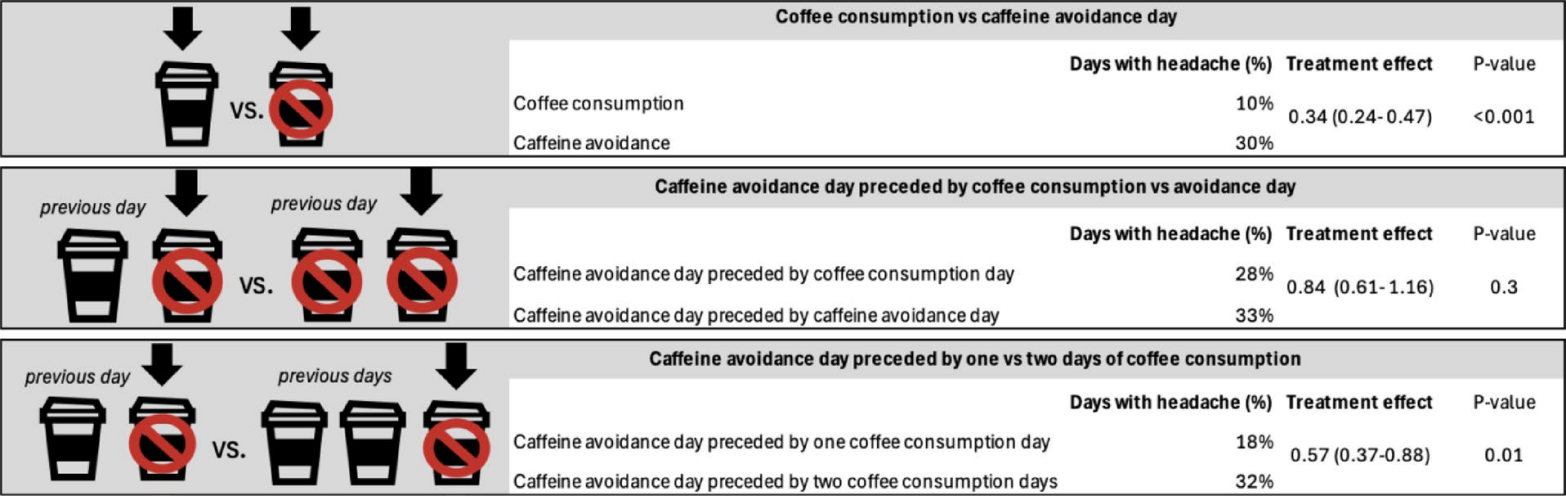
Headache Occurrence for Three Patterns of Caffeinated Coffee Consumption Based on Intention-to-Treat Data. Treatment effect estimates are adjusted for the day of the week and are shown as rate ratios.

76% of participants reported a headache during at least one day of study participation. Headaches occurred on a median of 2 (IQR 1–3) days across all participants, comprising 21% (IQR 7–33%) of study days. In crude analyses, a headache was reported on 10% of all days randomly assigned to caffeinated coffee and on 30% of all days randomly assigned to avoid all caffeine (Fig. 1). After adjusting for day of the week and after taking clustering within individuals into account, randomized assignment to abstain from caffeine was associated with twice the risk of a headache compared to caffeinated coffee consumption days (Fig. 2).

There was no significant difference in the frequency of headache occurrence on days when participants were randomized to abstain from caffeine after assignment to consume caffeinated coffee the previous day as compared to assignment to abstain from caffeine the previous day (Fig. 2, Supplementary Table S3). Additionally, headaches occurred significantly less frequently on days when participants were randomized to abstain from caffeine following two prior days of caffeinated coffee consumption as compared to following just one prior day of caffeinated coffee consumption (Fig. 2, Supplementary Table S4).

### Presence or absence of headache: as-treated

Every additional cup of caffeinated coffee consumed was associated with a 40% reduced risk of experiencing a headache (Supplementary Table S2). The as-treated analyses regarding patterns of caffeinated coffee consumption yielded the same results as the intention-to-treat analysis as described above (Supplementary Table S3, Supplementary Table S4).

### Presence or absence of headache: subgroup analyses

The relationship between caffeine withdrawal and headache did not significantly vary across genotype-determined caffeine-metabolizer categories (Supplementary Table S5). However, the reduced risk of headache in the presence of caffeinated coffee was significantly more pronounced among those who experienced baseline caffeine withdrawal headaches (Supplementary Table S6). The relationship between caffeine withdrawal on headache occurrence decreased with higher baseline caffeinated coffee consumption (Supplementary Table S7).

### Headache severity: intention-to-treat

In crude analyses, the median headache severity on days when a headache was present was 2 out of 10 (IQR 1–4). After adjusting for day of the week and after taking clustering within individuals into account, a headache (when present) was on average 73% less severe on days randomly assigned to caffeinated coffee versus caffeine avoidance (Supplementary Table S2). There was no significant difference in the severity of headache occurrence on days when participants were randomized to abstain from caffeine after being randomized to consume caffeinated coffee the previous day as compared to being randomized to abstain from caffeine coffee the previous day (Supplementary Table S3). When a headache was present, it was on average nearly 41% less severe on caffeine abstinence days following two prior days of caffeinated coffee abstinence as compared to following just one prior day of assignment to caffeine coffee consumption (Supplementary Table S4).

### Headache severity: as-treated

Each additional cup of caffeinated coffee was not significantly associated with reduced headache severity (Supplementary Table S2). The as-treated analysis regarding patterns of caffeinated coffee consumption also showed no significant differences in headache severity (Supplementary Table S3, Supplementary Table S4).

## Discussion

These data from a randomized case-crossover trial demonstrate that, among individuals regularly exposed to caffeinated coffee, caffeine avoidance was commonly associated with headaches. When headaches were present, caffeine avoidance also appeared to heighten the severity of the headache. These results suggest that headache, commonly experienced by a substantial proportion of the population, may often occur due to withdrawal of caffeine.

Headaches are widely experienced neurological conditions that can negatively impact productivity and quality of life^1,2,5–7^. Many headache triggers, however, remain largely uncharacterized^8^. While caffeinated coffee is regularly consumed by more than half of all American adults^17,18^, its actual health consequences remain largely informed by observational studies prone to confounding. Moreover, even among individuals that consume caffeinated coffee on a regular basis, interruptions are essentially inevitable, such as when people require medical procedures, during travel, or when they decide to make dietary changes^19^. Therefore, it is important to elucidate the effects of such interruptions and uncover readily modifiable strategies to prevent headache occurrence and reduce severity, such as by modulating one’s intake of caffeinated coffee.

These results build upon previous evidence from a crossover trial of 62 individuals undergoing a battery of tests in a study center showing that withdrawal from formulated capsules of caffeine was associated with a greater frequency of moderate or severe headache^16^. In contrast, the case-crossover CRAVE trial assessed commonly consumed caffeinated coffee specifically, and the outcome was ascertained while participants were ambulatory in the course of what was otherwise their normal lives. While the two studies are complementary and the main findings are consistent, the current study likely better recapitulates the actual circumstances of many in the general population.

Our results also highlight the potential impact of withdrawal timing on headache occurrence. For example, given evidence that the chance of caffeine avoidance-related headache was no different whether it occurred after a prior caffeinated coffee consumption day or avoidance day, it appears such withdrawal headaches may persist beyond just one day. In contrast, the significant reduction in headache occurrence on caffeine avoidance days that were preceded by two consecutive days of caffeinated coffee consumption versus just one day of caffeinated coffee consumption implies that consecutive days of caffeinated coffee may confer a resistance to the withdrawal headache associated with sudden caffeine cessation. This suggests that regularity and continuity in caffeinated coffee intake may mitigate the frequency and severity of headaches associated with the abrupt cessation of caffeine.

The apparent protective effect of caffeinated coffee consumption against headaches was stronger for those who experienced baseline caffeine withdrawal headaches and for individuals who drank fewer cups of caffeinated coffee per day at baseline. Interestingly, the speed of caffeine metabolism based on genotype did not appear to influence these relationships—while faster caffeine metabolizers might be expected to be more prone to caffeine-withdrawal headaches, perhaps the effect is so rapid as to occur even within the same day (requiring greater resolution than the current daylong-based randomization could provide).

Additionally, our results show that headaches, when present, appeared to be less severe on days randomly assigned to consume caffeinated coffee. This demonstrates that consuming a cup of caffeinated coffee not only reduces the likelihood that a headache will occur but can mitigate the possibility that a headache will reach a level of severity such that it impacts one’s quality of life or rises to clinical significance.

Our as-treated analysis demonstrated a dose-response relationship between the number of cups of caffeinated coffee consumed and headache occurrence, with each additional cup leading to reduced likelihood of experiencing a headache. However, each additional cup of caffeinated coffee was not significantly associated with reduced headache severity. This suggests that simply drinking any amount of caffeinated coffee can mitigate against withdrawal headaches—this finding may be useful for individuals deliberately titrating their caffeinated coffee intake, such as reducing it while traveling to adjust to facilitate sleep in the setting of time zone changes.

It is important to acknowledge limitations of this study. This trial was relatively small, enrolling 100 volunteers. However, the case-crossover design of the trial allowed for more than 1,000 assessments to be included in our analyses. This trial enrolled healthy volunteers, and the details of any headache-related history (such as a history of migraine headaches or use of caffeine-containing medications) were not elucidated. Indeed, this is best interpreted as an analysis of within-individual variability in headache symptoms. While we assume the associations between caffeinated coffee consumption versus avoidance of all caffeine and headache were due to caffeine, this study was not designed to determine the exact mechanisms underlying these observed relationships, particularly in participants who may have a history of migraine or another primary headache syndrome. The randomized structure of the data should mitigate against confounding, although we cannot exclude the possibility of some time-varying causal actor (that would have traveled with randomized instruction to consume versus avoid caffeinated coffee)—of note, the original CRAVE trial failed to demonstrate any differences in continuous glucose recordings, suggesting other dietary factors, such as related to sweeteners, were unlikely to be operative^18^. Participants were not blinded to the exposure, although this enabled a randomized trial in ambulatory individuals engaging in the full (and arguably “real-life”) experience of consuming or avoiding caffeinated coffee drinks. Additionally, although we examined patterns of coffee consumption across multi-day periods to account for short-term temporal dynamics related to caffeine withdrawal, these analyses may not fully capture the timing of peak withdrawal symptoms. We acknowledge that we did not collect daily information regarding medication use, such as intake of nonsteroidal anti-inflammatory drugs or acetaminophen, which could have affected both headache and withdrawal phenomenology. Finally, this study examines only the acute effects of caffeine withdrawal on headache occurrence and severity. Whether caffeinated coffee avoidance more chronically might reduce or increase the overall burden of headaches is worthy of future study, particularly given that long-term caffeine overconsumption may be associated with long-term headache and medication overuse headache^20,21^.

These findings may be useful in informing behaviors and recommendations regarding caffeinated coffee consumption and minimizing headache burden on a day-to-day basis. Specifically, if individuals who commonly consume coffee wish to mitigate headache symptoms associated with withdrawal, there may be benefit to avoiding sporadic days of caffeinated coffee abstinence. If one is looking to reduce total caffeine intake within a day, it may be reasonable to drink only one cup of caffeinated coffee. Patients and providers alike should consider the impact of caffeine withdrawal on headache when making or suggesting lifestyle adjustments or dietary changes.

## Methods

### Study population

This was a post hoc secondary analysis of the prospective, randomized, single-center, 14-day case-crossover Coffee and Real-time Atrial and Ventricular Ectopy (CRAVE) trial (ClinicalTrials.gov number NCT03671759, first registered14/09/2018), which has been previously described^18^. Primary and secondary outcomes were reported in the previous manuscript^18^. Study participants included 100 healthy adult volunteers. The trial was approved by the institutional review board at the University of California, San Francisco, and all participants provided written informed consent. All study procedures were conducted in accordance with applicable institutional guidelines and regulations. Exclusion criteria were (1) history of atrial fibrillation or heart failure; (2) individuals with an implanted pacemaker or implantable cardioverter–defibrillator; (3) individuals who had been prescribed beta-blockers, nondihydropyridine calcium-channel blockers or Vaughn–Williams class 1 or 3 antiarrhythmic medications; (4) having a medical reason to avoid coffee.

### Real-time assessment of coffee consumption and headache

Participants received random daily assignment to consume caffeinated coffee or to avoid all caffeine. Instructions were delivered via short message service (SMS) text at 8 pm the day prior, and a reminder text reinforcing their assignment was sent 8 AM the day of. Randomization was conducted in pairs of off-on or on–off days to ensure that a participant had no more than two consecutive days of coffee consumption or abstinence; this also enabled a maximum number of “switches” between assignments (yet still in a random order) in case of early withdrawal. Further, participants were fitted with a continuously recording electrocardiogram (ECG) patch (Zio XT Patch, iRhythm) and instructed to press an activator button on the ECG patch when they had consumed a standard (approximately 8-ounce) cup of caffeinated coffee or for every two standard (1-ounce) shots of espresso. This allowed a prospective assessment of every caffeinated coffee drink. This process has been previously validated for real-time assessment of alcoholic drink consumption^22^. This method was also previously shown to be accurate in reflecting caffeinated coffee in CRAVE using three methods of compliance ascertainment: daily surveys delivered via text that assessed the number of coffee drinks consumed the prior day; participants provided date-stamped receipts for caffeinated coffee purchased for immediate consumption (they were incentivized to do this given notification prior to study initiation that all caffeinated coffee accompanying a data-stamped receipt would be reimbursed by the study, whether they were randomly assigned to consume or avoid caffeine that day); and “geofencing” coffee shop visits were also correlated^18^.

Every evening (at 8 pm local time), participants were asked via an SMS text-based survey to report whether they were experiencing a headache at that moment and, if present, to rate the severity on a scale from 1 to 10. A score of 1 represented the lowest severity and 10 represented the most severe headache. Headache was a self-reported outcome and was not subclassified into diagnostic categories such as migraine or tension-type headache.

### DNA sample collection and analysis

DNA was collected and purified from saliva and genotyped for seven caffeine-related, single nucleotide polymorphisms with the use of a real-time polymerase-chain-reaction assay in order to determine participants’ caffeine metabolism-related genotype score (TaqMan Master Mix and Applied Biosystems 7300)^23–25^.

### Statistical analysis

Normally distributed continuous variables are described as means ± SD and compared using t-tests; continuous variables with skewed distributions are described as medians and interquartile ranges (IQR) and were compared using the Wilcoxon rank sum tests. Categorical variables were compared using the chi-squared test; Fisher’s exact tests were used when observed cell counts were less than five. Headache occurrence and severity outcomes were analyzed using log-Poisson generalized linear mixed models (also to account for within-participant clustering) adjusted for the day of the week, with results reported as risk reduction (RR). Analyses examining associations within strata of baseline headache status, baseline coffee consumption, and polygenic caffeine-metabolizer categories employed logistic models to account for clustering within participants, with results reported as odds ratios (OR). Interaction testing was performed using generalized linear mixed models. The primary analyses utilized the intention-to-treat principle using randomization assignment as the primary predictor. As-treated effects were also assessed based on actual caffeinated coffee consumption as recorded in real-time by participants pressing the wearable activator button. While headache data was prospectively collected on a daily basis as described in the original research protocol, the specific methods of analyzing those results were not pre-specified at the time the study was designed. Three patterns of caffeinated coffee consumption in relation to headache occurrence and severity were analyzed: (1) caffeinated coffee consumption versus caffeine avoidance days; (2) caffeine avoidance days preceded by a caffeinated coffee consumption versus avoidance day; and (3) caffeine avoidance days preceded by one versus two days of caffeinated coffee consumption. Study duration was determined by the number of days participants completed headache survey questionnaires.

## Supplementary Information

Below is the link to the electronic supplementary material.

Supplementary Material 1

Supplementary Material 2

## Data Availability

A deidentified dataset will be provided upon reasonable request to the corresponding author.
