# Supplementary material for: Acute effects of caffeine withdrawal on headache among regular caffeinated coffee drinkers
Source: Sci Rep. 2026 May 22;16:23299. doi: 10.1038/s41598-026-54049-3 (PMC13402579; doi:10.1038/s41598-026-54049-3)
Supplement: Supplementary file 1 — Supplementary Material 1 [file 41598_2026_54049_MOESM1_ESM.pdf]

CRAVE Study Protocol

Authors: Gregory M. Marcus, MD, MAS
Principal Investigator, UCSF Division of Cardiology

Christina Fang
Clinical Research Coordinators, UCSF Division of Cardiology

Version 1

Date: July 19, 2018

1.0 Introduction

1.1 Background

Atrial fibrillation (AF) and heart failure (HF) are morbid, costly, and incompletely understood diseases that have reached epidemic proportions worldwide. A dose-dependent relationship exists between premature atrial and ventricular contractions (PACs and PVCs) and development of AF and HF, respectively. Identifying and understanding the mechanisms of additional modifiable risk factors for ectopy has the potential to markedly reduce the healthcare burden of these diseases. In considering how we might modify the prevalence of these ectopic beats, common exposures in daily life are prime candidates. Current guidelines suggest that caffeine may be an important trigger for frequent ectopy, although large population-based studies have not demonstrated an association between caffeine consumption and development of clinically significant arrhythmias. No study has employed an actual randomization intervention to assess the effects of caffeine on cardiac ectopy. Utilizing the Eureka platform, we plan to utilize the N-of-1 strategy to rigorously investigate the real-time effect of caffeine intake on ectopy.

1.2. Study Design

The study will rigorously investigate the real-time effect of caffeine intake on ectopy, and we hypothesize that patients will have more ectopy on days when they consume coffee compared to days without coffee consumption. We aim to enroll 80 participants into this study, with the goal of elucidating the relationship between caffeine consumption and heart rhythm changes. In order to do this, participants will wear an automatically recording electrocardiographic monitor, continuous glucose monitor, and utilize Eureka—a mobile application—as well as a fitness tracker, over a two week period. We will compare participant self-

report of caffeine consumption, and heart rhythm data from the event monitor in order to better understand the relationship between acute caffeine consumption and heart rhythm changes.

2.0 Study Devices:

2.1 Zio XT Patch

Overview

The ZIO® XT Patch is a continuously recording, wire-free heart monitor that can be worn for up to 14 days. Every beat is recorded on the device, which is mailed in a prepaid box back to iRhythm for analysis. iRhythm will not receive any patient identifying information. Zio XT Patch reports must be re-identified prior to uploading to the patient's electronic medical record.

Ordering Devices

Patches are ordered through the STUDY-EXPOSURES UCSF account charged to the following speedtype: **MMEPCA18GM**. The patches are ordered through a Consignment system, in which the study coordinator will request patches from Karlene Lee-Tung (kleetung@irhythmtech.com) and copy Lori Crosson (lcrosson@irhythmtech.com). Please reference the correct PO **B001404269** to invoice the correct speedtype.

Device Data

Reports are posted on iRhythm Reports (https://www.zioreports.com/Application.html#HOME) and will include the information contained in a standard report as well as daily ectopy burdens, per the Addendum A-2 Statement of Work for iRhythm Technologies, Inc. Research established under STUDY-EXPOSURES UCSF. iRhythm will also provide exports of data from each patch including episodes of AF greater than 30 seconds, SVT greater than 4 beats, and VT greater than 4 beats on a monthly basis. Finally, iRhythm will also provide raw data files (MIT files) from the ZIO® XT Patch through UCSF Box. Please contact Karlene Lee-Tung (kleetung@irhythmtech.com) for updated PAC/PVC reports and episodic data; please contact George Sarlas (gsarlas@irhythmtech.com) to request MIT files.

Upon participant request, we will also share individual-level results collected from the Ziopatch, and provide reports showing randomizations over the enrollment period (dates randomized to consume caffeine vs abstain from caffeine). Should any significant clinical findings be discovered from the Ziopatch reports, a UCSF Cardiologist will review the reports and contact the participant directly, as well as potentially follow up with the participant's primary care physician. Participants will consent to disclose findings with their primary care physician at the discretion of the investigator when participants consent to the study.

2.2 FitBit

Overview

The FitBit device used for this study is the Fitbit Inspire (without HR information). We will primarily be collecting data on step count and hours slept. Data from these devices will be shared with the study team as participants agree to connect their Fitbit device through Health eHeart, which as a tenant of the Eureka study, will link all relevant participant data to the CRAVE study.

Ordering Devices

We submitted a Fitbit Research Application which was approved March 28th, 2019, qualifying our study for research discounted Fitbits ($55.95/each). These devices must be ordered in quantities of 30+, or else will

be priced at retail value ($69.95/each). Order devices through Bearbuy and invoice them to **MMEPCA18GM**; search on Bearbuy (Under "Owner: Christina Fang; Supplier: Fitbit") for example of placed orders and see Appendix B for more detailed instructions on ordering.

Device Data
Data from Fitbit will be monitored by the Health eHeart team. In order to request this dataset, please reach out to Health eHeart data extraordinaire David Wen (David.Wen@ucsf.edu), and copy Emina Seremet (Emina.Seremet@ucsf.edu).

2.3 Dexcom G6

Overview
The Dexcom G6 is an FDA approved continuous glucose monitor (CGM). They are painless and adhere to the skin; CGM users insert a tiny sensor wire just under their skin using an automatic applicator. An adhesive patch holds the CGM sensor housing in place so the sensor can measure glucose readings throughout the day and night. The monitor measures glucose information in the interstitial fluid, which is a thin layer of fluid surrounding cells right below the skin, and collects glucose information every five minutes.

Ordering Devices
Fill out the "TEMPLATE External Study Order Form" in CRAVE-> Correspondence -> Dexcom Order Forms, and email the completed form to clinicalorders@dexcom.com. Each person will receive a single transmitter and a single sensor, and we will not be collecting these devices.

Device Data
To set up a new Dexcom G6 user, please follow the following steps

1. CRC: Create new user at Dexcom clarity with "### XYZ", following the steps outlined above
2. Participant: Download Dexcom G6 app on participant phone
3. Participant: Sign up for account
4. Participant: Confirm email
5. Participant: Create log in
6. Participant: Log in and complete set up of transmitter on Dexcom app
7. Participant: Go to: https://clarity.dexcom.com/ or simply stay in the app
8. Participant: Go to Settings or if in the app, "Share data"
9. Participant: Scroll down to "Data Sharing with Clinics"
10. Participant: Click "+Share Data with a New Clinic"
11. Participant: Enter sharing code generated by CRC

Dexcom Clarity
Data collected by the Dexcom G6 will be populated on Dexcom Clairty, under the "UCSF CRAVE Study" Clarity Clinic. To access the Clinic, please go to: https://clarity.dexcom.com/professional/ and enter the following log in information:
User: ucsfcrave
Pass: Biospecimen1!

To add a new participant, click on "Add new patient", enter the Study ID for first and last name, as well as the real date of birth. Once the participant is created, click on the name and opt to "Share data" at the bottom right hand corner.

For more detailed instructions on device administration and Clarity account set up, please refer to “Dexcom G6 Training.pdf”, “Dexcom Clarity.pdf” in the “CRAVE Study Arm” -> Protocol -> Dexcom.

3.0 Eureka Mobile Application

3.1 Overview of Eureka

We will use the Eureka mHealth Research Platform (CHR #16-19397) to facilitate single subject (N-of-1) experiments designed to answer key participant-identified research questions about personalized caffeine use.

3.2 FitBit API

In order to connect your Fitbit data to Health eHeart, and by extension Eureka, participants will be required to sign up for a Health eHeart account (https://www.health-eheartstudy.org/). Once the account is set up, direct participants to “Profile”, and “HIPAA Consent” under Medical History. Complete the HIPAA Consent form on Docusign.

Next, select “Fitbit” under “Connected Devices”. You will be navigated to log into Fitbit.

If the participant already owns a Fitbit, please have them log in with their Fitbit login. Once you are logged in, select “Allow All”, or as participants are comfortable.

If the participant does not yet own a Fitbit, please have them sign up for an account and log in. Once you are logged in, select “Allow All”, or make the selections that the participant is comfortable with.

Next, you must link your Fitbit device with your Fitbit account. To do so, you will need to download the Fitbit app on your phone.

Afterwards, Log in with your Fitbit log in information.

Select the Menu icon at the upper right hand corner, and click “Set Up a Device”. Select “Inspire”, and follow the instructions on the phone screen and Fitbit device to pair the device.

3.3 Geofencing Feature

Have participants go to: https://eureka.app.link/cravetest from their phone and enroll in the Geofencing application.

4.0 Mosio Text Messaging Service

4.1 Overview

Mosio a text messaging software for clinical research. Mosio will be able to deliver daily text reminders with surveys.

The Master File containing the randomization scheme and REDCap unique survey links is in the Shared Drive (Monitors → CRAVE Study Arm → Participants→ CRAVE_Master File.xlsx). Only make edits to the

green columns. It is very important not to alter the randomization column or the survey link columns, as these were generated specifically for each study ID.

4.2 Enrolling New Participant

When there is a new participant, fill in the "phone number", "ppt_id", and "start date" in the CRAVE_Master File.xlsx and save the file.

Next, copy the row corresponding to the new participant ONLY. This is very important; each time you upload a new participant to Mosio, duplicate phone numbers will be reset and people will start over. Paste the entire row into a new Excel file and save it as a CSV file in the participant's folder, with the file name "*ppt id_ppt initials*_Randomization".

Next, go to: https://biz.mosio.com/login?return=https://biz.mosio.com/par/mb/view/ucsfch

1. Log in with user: cfangadmin, password: Biospecimen1
2. Under Quick Links, click on "UCSFCH Uploader"
3. Click "Choose File"
4. Select "*ppt id_ppt initials*_Randomization".csv file from the participant's folder
5. Click "Upload CSV and Enroll"
6. To confirm that the participant has been enrolled, he/she should immediately receive a text welcoming them to the CRAVE study.

4.3 Checking Participants

Click "Contacts", "ucsfch"

To look at Texts sent, go to "Actions"→ "Text History" and you should see all outbound texts.
To look at Randomization Calendar, go to "Actions"→ "Participant Profile" → "Personal Storylines" and you should see all texts to be sent, in line view as well as Calendar view. You are able to confirm the correct messages will be sent through either view, and also make adjustments to time of message if needed. Otherwise, messages will be sent at 8:00am and 8:00 pm.
To remove a participant, go to "Actions"→ "Remove from Contacts"

4.4 Randomization Scheme

128 unique combinations of A & B were generated, and each was assigned a number between 1-128. Numbers 1-128 were randomized; the new order of number corresponds to the randomization scheme for each additional enrolled participant. I.e., the new order could be: 48, 19, 52, 102, 3, etc. This means participant #1 will receive randomization scheme 48, participant #2 will receive randomization scheme #19, and so on. If participants early terminate, the next participant will take up the next available number. If/when all 128 numbers are exhausted, numbers 1-128 are re-randomized into a new order, and the next participant will take on the first available randomization.

5.0 REDCap

5.1 Database

The database has been set up to accommodate 128 participants. The survey links are unique to each survey. As much as possible, do not make edits to the "Morning Check-In" and "Evening Check-In" surveys, as this might generate new survey links and will require massively updating the Master File, which otherwise would not need to be edited.

Please feel free to edit the “Receipt Information” and Enrollment surveys as needed, as this will not influence the survey links.

To retrieve survey links, go to “Survey Distribution Tools”, select the survey you would like to generate links for under “Participant List belonging to ___________” and select “Export list”. The survey links generated in Column G correspond are those that populate the Master File.

The specific number of repeated survey instruments has also been adjusted to accommodate CRAVE; to make any edits (not recommended), go to “Project Set Up”, “Designate Instruments for My Events”, and “Begin Editing”.

5.2 Adherence

Check the record status dashboard daily to see if there are participants who need a gentle reminder to complete their surveys. Ideally, we will not be missing more than 3 days of data in a row.

6.0 Genetic Sample

6.1 DNA Genotek Orangene DNA

We will be collecting saliva samples utilizing the DNA Genotek Orangene DNA spit kit. The kit collects 2 mL of saliva excluding any volume from air bubbles in saliva. Please advise patients to avoid drinking or eating during the 30 minutes leading up to sample collection. To order spit kits, contact Kasia. For samples to be processed, drop samples off at Mission Bay with Emily Wilson and request for her to complete processing.

Once participants have consented to participate in CRAVE, the study coordinator will add each new subject in OnCore under Menu -> Specimens -> Specimen Collection Console -> New Patient.

Upon participant request, we can provide individual-level results collected from the genotyping. However, we will inform participants that we will only be sharing information regarding caffeine metabolism that bears no clinical significance and only represents results from this research study (as opposed to clinical care).

7.0 Eligibility

7.1 Inclusion Criteria

1. Are age 18 or older
2. Have a smartphone
3. Are able to use the Eureka mobile application
4. Are willing to provide a saliva sample for genetic processing
5. Drink coffee or coffee-based products at least once a year
6. Are willing to abstain from coffee, caffeinated products, or minimally caffeinated products (decaffeinated coffee) for at least 2 days when instructed

7.2 Exclusion Criteria

1. Have a history of atrial fibrillation, heart failure

2. Have an ICD or pacemaker
3. Have treatment with beta blockers, non-dihydropyradine calcium channel blockers, or Vaughn-Williams class 1 or 3 antiarrhythmic medications
4. Have a medical reason to avoid coffee

8.0 Participant Recruitment

8.1 Participant Recruitment

Prospective candidates will be recruited through advertisements (i.e. IRB-approved posters and business cards), by word of mouth, or referral from medical or research staff. Potential study candidates will reach out to study staff at the number (415) 476-4999, or directly email the study coordinators. Over the phone or email, study staff will confirm that the participant meets eligibility criteria before scheduling participant for a visit at Parnassus or Mission Bay. Informed consent will be obtained via Eureka prior to any study related procedures; the study participant will retain all consent documents through Eureka. Study participation is anticipated to be 14 days with two visits occurring at enrollment and week 2. The participant may withdraw voluntary participation at any time. An early termination visit should be done in the event that a participant chooses to discontinue participation prior to study completion.

8.2 Participant Reimbursement

Study participants will be fully reimbursed for their study-related purchases, upon proof with time-stamped receipts. Participants will be asked to track purchases through Eureka, keeping photographic record of receipts. Per IRB# 18-25195, acceptable forms of payment are cash, check, gift card, and debit card. For petty cash reimbursements, study staff must be an authorized petty cash custodian; the procedure to set up a petty cash fund can be found here: https://controller.ucsf.edu/how-to-guides/accounting-reporting/cash-handling-credit-cards/petty-cash-procedures.

8.3 Participant Quotas

We need 80 patients, so, assuming pretty much everyone will be between age 18 and around 80, we were thinking of constraining enrollment this way:
Age 18-19, n=13
age 20-29, n=13
age 30-39, n=13
age 40-49, n=14
age 50-59, n=14
age 60-69, n=13
age 70+, n=13

9.0 Study Procedures

The 2-week visit should be **after** 14 days, and not the 14th day. , and the 4-week visit should be at 28 ±3 days. Participation can be truncated to a 21-day minimum for patients with a scheduled ablation within the 28-day time frame.

9.1 Enrollment Visit

The Enrollment Visit takes place on Day 0; participants are still free to consume caffeine per their typical habits the day that they enroll. Participants will receive their first baseline survey this evening at 20:00.

At the Enrollment Visit participants will consent for CRAVE; following, participants will download the Geofencing feature, consent for Biospecimen, and connect their Fitbit. After all the consents are signed, the study coordinator will collect saliva.

Following, the study coordinator will ask the baseline surveys.

Prep:
1. Folder (paper consent, Redcap Q's, Reimbursement form)
2. Have Redcap open
3. Have Masterfile open
4. Have Mosio upload site open
5. Have Ziopatch
6. Have Spitkit
7. Have Fitbit
8. Have Dexcom

Visit Activities:
1. Paper consent participant
2. Answer RedCap questions
3. Enroll in Eureka for geofencing (https://eureka.app.link/cravetest)
4. Enroll in Health eHeart (https://www.health-eheartstudy.org/)
5. Sign Biospecimen Consent
6. Connect Fitbit
7. Set up Dexcom; download G6 app and set up Clarity (https://clarity.dexcom.com/)
8. Put on Dexcom
9. Input and upload number for Mosio (https://biz.mosio.com/login?return=https://biz.mosio.com/par/mb/view/ucsfch)`
10. Put on Zio
11. Collect spit
12. Reminders: Zio no swimming/bathing, Fitbit keep charged and Bluetooth on, Phone keep location services on

After Visit:
1. Register Ziopatch

9.2 Monitoring Period

The monitoring period is Day 1-14. Participants will be receiving daily instructions at 08:00 and reminders at 20:00, as well as their daily surveys.

The AM message includes instructions for the day, and daily surveys for caffeine use and sleep.

The PM message includes instructions for the next day, and daily surveys for mood and headaches.

After Day 14, participants are welcome to resume their normal caffeine habits, and will be prompted to do so by the PM message.

Participants will receive their final message on Day 15, to finish their last set of surveys and will be

reminded to resume their normal caffeine habits.

9.3 Termination Visit

The Termination Visit can be scheduled any day after Day 14. At the Termination Visit, collect back the Ziopatch and drop it off in USPS mail.

Prep:

1. Have folder
2. Bring Zio box

Visit Activities:

1. Collect back Zio
2. Collect receipts
3. Fill out reimbursement

9.4 Early Discontinuation

Early Discontinuation/Termination

If a participant wishes to discontinue participation, collect study devices and conduct an early termination visit if the participant is agreeable. Participants can be removed as a contact by “removing contact”, as above, or text “stop” to the CRAVE texts.

Table 1. Schedule of Procedures

Schedule of Procedures		
	**Enrollment**	**Termination**
Informed Consent	X	
REDCap Questions	X	
Geofencing (Eureka)	X	
Health eHeart Enrollment	X	
Biospecimen Consent	X	
Connect Fitbit	X	
Set Up Dexcom	X	
Administer Dexcom	X	
Upload Number for Mosio	X	
Administer Zio	X	
Collect Saliva	X	
File Reimbursement		X

APPENDIX A Study Contacts

University of California, San Francisco	
Gregory Marcus, MD<br><br>Principal Investigator<br><br>marcusg@medicine.ucsf.edu<br><br>Direct: (415) 476-3450<br><br>EP Office: (415) 476-5706<br><br>505 Parnassus Ave, M1180B, Box 0124	Christina Fang<br><br>Clinical Research Coordinator<br><br>Christina.fang@ucsf.edu<br><br>Direct: (415)476-4999<br><br>505 Parnassus Ave, M1180, Box 0124
David Rosenthal<br><br>Research Investigator<br><br>David.rosenthal@ucsf.edu	Kelsey Ogomori<br><br>Clinical Research Coordinator<br><br>Kelsey.ogomori@ucsf.edu<br><br>Direct: (415) 502-3053<br><br>505 Parnassus Ave, M1180, Box 0124
	Defne Yilmaz<br><br>Clinical Research Coordinator<br><br>Defne.Yilmaz@ucsf.edu<br><br>Direct: (415) 502-3489<br><br>505 Parnassus Ave, M1178, Box 0124

The Coffee and Real-time Atrial and Ventricular Ectopy (CRAVE) Trial

Authors: Gregory M. Marcus, MD, MAS
Principal Investigator, UCSF Division of Cardiology

Kathleen Chang, Michelle Yang, Grace Wall
Clinical Research Coordinators, UCSF Division of Cardiology

Version 2

Date: 8/24/2021

1.0 Introduction 4
1.1 Background and Preliminary Results 4
1.2 Significance 4

2.0 Investigational Plan 5
2.1 Study Design 5
2.1.1 Study Visits and Windows 5
2.2 Study Objectives 6
2.3 Inclusion/Exclusion Criteria 7
2.3.1 Inclusion Criteria 7
2.3.2 Exclusion Criteria 7

3.0 Study Treatment 7

4.0 Follow up Status 9
4.0.1 Early Termination: Discontinuation of Follow-Up 9

5.0 Study Procedures 9
5.0.1 Participant Recruitment 10
5.0.2 Eureka Mobile Application: 10
5.0.3 ZIO® XT Patch Event Monitor: 11
5.0.4 Fitness Trackers: FitBit INSPIRE Tracker 11
5.0.5 Dexcom G6 11
5.0.6 Biospecimen Collection: 12
5.0.7 Geofencing Feature: 12
5.0.8. Mosio Text Messaging Service 12
5.1 Enrollment Visit 12
5.2 Monitoring Period 13
5.3 Termination Visit 13
5.4 Early Discontinuation 14

6.0 Risks and Benefits of the Study Procedure 14
6.1 Potential risks: 14
6.2 Potential benefits: 14

7.0 Study Roles 15

8.0 Protocol Deviations 15

9.0 Quality Insurance and Data Management 15
9.1 Clinical Site Investigator and Coordinator Training 15
9.2 Data Handling and Confidentiality 15
9.3 Data Collection and Management 16

10.0 Statistical Methods 17

10.1 Sample Size and Randomization 17
10.3 Statistical Analysis Plan (SAP) 17

11.0 Ethical Considerations 18
11.1 Institutional Review Board (IRB) and Ethics Committee (EC) Approval 18
11.2 Informed Consent 18
11.3 Declaration of Helsinki 18

12.0 Protocol Amendments 18

Bibliography 20

1.0 Introduction

Atrial fibrillation (AF) and heart failure (HF) are morbid, costly, and incompletely understood diseases that have reached epidemic proportions worldwide. Previous studies have shown a dose-dependent relationship between premature atrial and ventricular contractions (PACs and PVCs) and development of AF and HF, respectively. Identifying and understanding the mechanisms of additional modifiable risk factors for ectopy has the potential to markedly reduce the healthcare burden of these diseases. In considering how we might modify the prevalence of these ectopic beats, common exposures in daily life, such as caffeine, are prime candidates.

1.1 Background and Preliminary Results

Premature cardiac contractions (PCCs), otherwise known as atrial and ventricular ectopy, are common throughout the general population (1, 2, 3), with recent and growing evidence showing strong associations between PCCs and several cardiovascular diseases. For example, studies have found PAC count us a useful predictor of incident AF in older adults (4) and increased PACs in healthy individuals can be associated with incident atrial fibrillation (AF), stroke, and death (5). PVCs are associated with an increased risk of incident congestive heart failure (CHF), coronary artery disease (CAD) events, and CAD-related death (6, 7).

However, little is known about modifiable exposures that may reduce or prevent frequent PACs or PVCs. While patients often associate the symptoms of ectopy with emotional stress, physical activity, dietary factors, and caffeine or other stimulant use (8), there is little data to support the role of behavioral modifications or trigger avoidance in reducing or preventing premature cardiac contractions.

We previously performed an analysis of more than one thousand community dwelling individuals in the Cardiovascular Health Study, selected to undergo 24-hour Holter monitoring (9), but we were unable to demonstrate a relationship between caffeine consumption and premature cardiac ectopies. However, that study had several important limitations we hope to address in the current study. First, coffee consumption was categorized only as the number of days consumed rather than amount consumed. Second, the study was observational and potentially affected by influential confounders. Third, the 24 hour Holter monitoring period was too short to analyze how daily ectopy levels may change with acute coffee consumption. This study’s current N-of-1 design, with randomized assignments to consume or abstain from coffee and a 14 day monitoring period, should help minimize influential confounders and improve our understanding on the real-time effects of caffeine consumption.

1.2 Significance

Despite being the most consumed beverage in the world, the immediate physiological effects of coffee remain unclear. Caffeine’s ability to stimulate the sympathetic nervous system, increase epinephrine and norepinephrine levels, and thus potentially increase ectopy is of particular interest. While current guidelines suggest that caffeine may be an important trigger for frequent

PACs and PVCs (10), large population-based studies so far have not been able to demonstrate an association between caffeine consumption and development of clinically significant arrhythmias.

The majority of coffee-related research has been observation and prone to confounding variables. Many examine long-term effects of caffeine less relevant to the immediate consequences. In fact, very few studies so far have employed randomization intervention to assess the real-time influence of caffeine on cardiac ectopy.

Through randomization intervention to either consume or abstain from coffee within an individual's monitoring period, this study will improve our understanding on the direct consequences of caffeine intake on physiological conditions, such as ectopy levels, physical activity, glucose levels, and sleep quality/duration. Furthermore, we will provide further insight into how genetically-determine differences in caffeine metabolism may influence these relationships.

2.0 Investigational Plan

2.1 Study Design

This study is designed as a N to 1 randomized trial, where each participant will be randomized to consume or abstain from coffee each day of a 14 day monitoring period. Participants will wear an automatically recording electrocardiographic monitor (ZIO® XT Patch), continuous glucose monitor (Dexcom G6), and a fitness tracker (Fitbit INSPIRE) while utilizing Eureka—a mobile application. We will compare participant self-report of caffeine consumption, glucose monitoring, fitness levels, sleep health, and heart rhythm data in order to better understand the relationship between acute caffeine consumption and heart rhythm changes. Participants will also be genotyped for caffeine metabolism-related SNPs. A total of 100 participants will be enrolled at the UCSF study site.

2.1.1 Study Visits and Windows

After screening and eligibility has been determined, participants will officially enroll through the Eureka app, receive study materials, and donate saliva for genetic analysis at the Baseline visit (Day 0). The participants will be in contact with study coordinators throughout the monitoring period, which starts Day 1 through 14. At the termination visit (Day 15), participants will return the heart monitor and devices to the research team. Figure 1 below outlines the study flow.

Figure 1. Study Flow Diagram

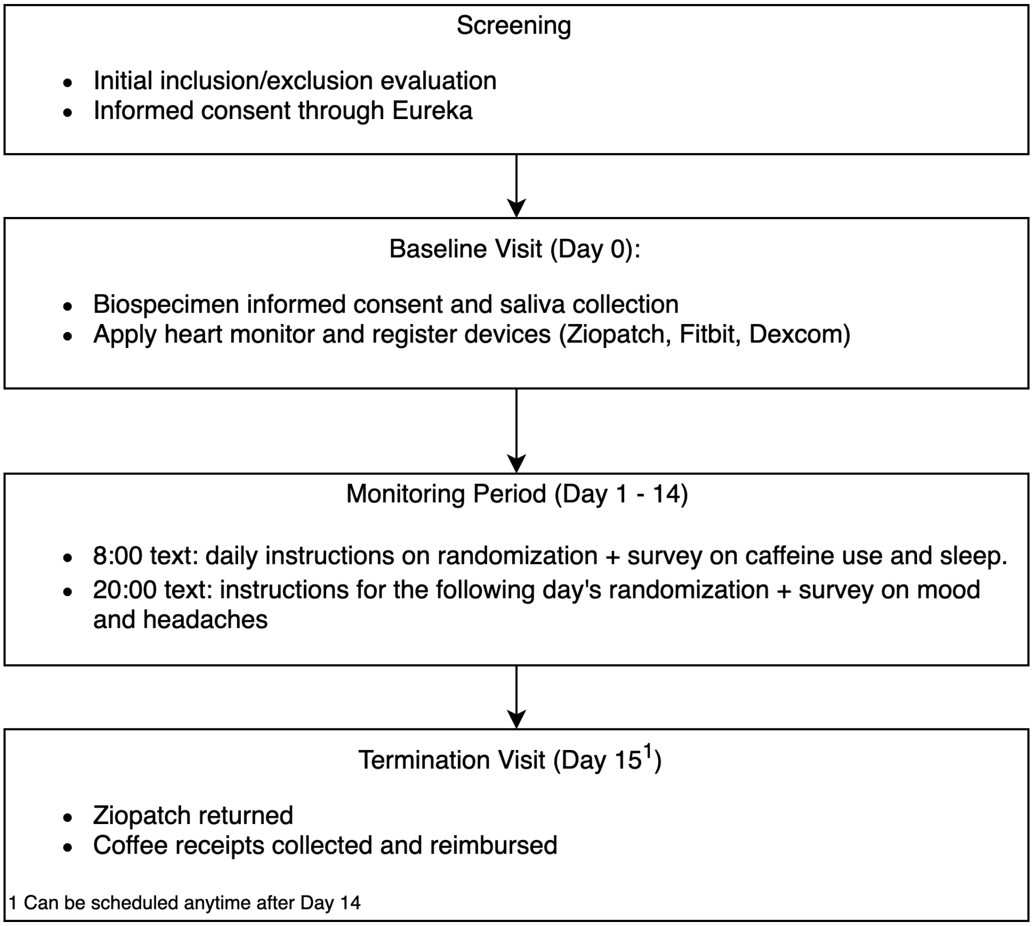

2.2 Study Objectives

The objectives of this trial are to study the real-time physiological consequences of caffeine consumption. We aim to specifically elucidate how caffeine affects glucose levels, sleep quality, physical activity, and daily ectopy on a day-to-day basis.

Primary objectives:

- To compare the changes in PACs and PVCs on days where participants were randomized to drink coffee vs. randomized to avoid caffeine
- To analyze the relationship between caffeine intake and general health measurements, ie. physical activity and sleep health

Secondary exploratory objectives include assessment of the following:

- To assess if physical activity, daily glucose levels, and sleep mediate coffee-ectopy relationships
- To assess if genetically-determine differences in caffeine metabolism influence the aforementioned associations

2.3 Inclusion/Exclusion Criteria

2.3.1 Inclusion Criteria

Patients must meet all of the following to eligible:

1. Are age 18 or older
2. Have a smartphone
3. Are able to use the Eureka mobile application
4. Are willing to provide a saliva sample for genetic processing
5. Drink coffee or coffee-based products at least once a year
6. Are willing to abstain from coffee, caffeinated products, or minimally caffeinated products (decaffeinated coffee) for at least 2 days when instructed

2.3.2 Exclusion Criteria

Patients will be excluded if they meet any of the following:

1. Have a history of atrial fibrillation, heart failure
2. Have an ICD or pacemaker
3. Have treatment with beta blockers, non-dihydropyridine calcium channel blockers, or Vaughn-Williams class 1 or 3 antiarrhythmic medications
4. Have a medical reason to avoid coffee

3.0 Study Treatment

During the initial visit, the study staff (i.e. study coordinator) will meet the study participant at UCSF (i.e. in the Clinical Research Center) or remotely through a phone call. Once study staff has determined that the participant is eligible for the study per the study's inclusion and exclusion criteria, the study staff will proceed with obtaining informed consent. After the participant has consented to participate in the study, the study staff will ask the participant questions to assess his/her caffeine habits (baseline caffeine intake survey, caffeine withdrawal symptoms survey), palpitations symptoms (Health eHeart palpitations survey), demographics, and medical history, as well as conduct a medical chart review. Afterwards, the study staff will explain the Eureka mobile application and relevant risks pertaining to loss of privacy (see Section 11.2). The study staff will instruct the participant how to download and set up the Eureka mobile application to the participant's phone, and also enable the relevant features (i.e. the geofencing feature). In the event that the participant is not amenable to downloading Eureka or it is non-functional, we will utilize the Mosio software and RANDOM.org to execute the needs of our study. Next, the study staff will explain the external remote cardiac monitor (i.e. ZIO® XT Patch), continuous glucose monitor (i.e. Dexcom G6), and relevant risks pertaining to potential skin irritation from the adhesive on the device. The study staff, all of whom are trained, will administer the device and instruct the participant on device care. The study staff will then explain that the purpose of the biospecimen collection is to investigate single nucleotide polymorphisms previously shown to affect caffeine metabolism. Following, the study staff will

collect a saliva sample from the participant utilizing the DNA genotek spitkit and explain relevant risks pertaining to loss of privacy (see Section 11.2). Blood sampling will only be conducted if participants elect to do a blood draw instead of saliva collection. All blood draws will be completed by trained medical staff. Upon participant request, study staff can provide individual-level results collected from the genotyping. However, the study staff will inform participants that the study staff will only be sharing information regarding caffeine metabolism that bears no clinical significance and only represents results from this research study (as opposed to clinical care). Finally, the study staff will give the participant a wrist-worn fitness tracker (i.e. Fitbit INSPIREdevice) and explain relevant risks as pertaining to potential discomfort from wearing the device and loss of privacy (see Section 12.1). The study staff will instruct the participant on device care. After the study staff has completed the device education and fitting, the study staff will schedule the participant's final visit.

During the study, patients will receive a text or push notification from the Eureka app or Mosio software each day with instructions for allowance or restriction of coffee intake the following day and will also receive daily queries regarding consumption that occurred the previous day. A cup of coffee will be defined as one 8 oz coffee (130-180mg caffeine, depending on roast), or two espresso shots (150-170mg caffeine). In order to avoid >2 consecutive days with or without coffee, participants will be randomized in pairs of either "off one day and on the next day" or "on one day and off the next day". All participants will be instructed to press the button on the ZIO® XT Patch whenever they have a cup of coffee. All patients will also receive a Fitbit Inspire to assess step counts and sleep. Patients will "opt in" to share their data with Eureka via an API (Application Programming Interface, see more details in Section 12.1). Participants will consent by clicking an "opt in" web form from the Eureka platform, and then complete the opt-in procedure specified and managed by each third party. Participants who do not download Eureka will consent to share their fitness tracker information with the study team through Health eHeart (CHR #12-09993) in Module 5. In addition, we will be monitoring how glucose levels fluctuate with changing caffeine habits, thus participants will also be given a continuous glucose monitor to wear for the duration of the study.

During the final visit, the study staff (i.e. study coordinator) will meet the study participant at UCSF (i.e. in the Clinical Research Center) at the time specified by the study participant. Next, the study staff will remove the external remote cardiac monitor (i.e. ZIO® XT Patch) and continuous glucose monitor (i.e. Dexcom G6). Upon participant request, study staff can provide individual-level results collected from the device and provide reports showing randomizations over the enrollment period (dates randomized to consume caffeine vs abstain from caffeine). The study staff will then collect back the fitness tracker (i.e. Fitbit INSPIRE device). Once all equipment has been returned, the study staff will assess the participant's study compliance and conduct quality checks for equipment data collection. At this time, the study staff will also reimburse the participant for all caffeine purchases upon proof with time-stamped receipts.

4.0 Follow up Status

If a randomized participant terminates participation in the study early (i.e., prior to completion of all follow-up), the clinical site Principal Investigator must determine the primary reason for early termination and report this. Participants who early terminate from the study or do not complete all study measures will be replaced with newly enrolled participants such that the total participants enrolled who complete all study measures is 80.

4.0.1 Early Termination: Discontinuation of Follow-Up

Each participant will be informed that participation in the study is voluntary and that he/she may withdraw from the study at any time without effect on subsequent medical treatment or relationship with the treating physician. Participants who discontinue follow-up at any time after randomization will be included in the ITT analyses.

5.0 Study Procedures

Table 1 provides an overview of the evaluations to be performed at each of the study visits.

Table 1. Schedule of Procedures

**Schedule of Procedures**		
	**Enrollment**	**Termination**
Informed Consent	X	
REDCap Questions	X	
Geofencing (Eureka)	X	
Health eHeart Enrollment	X	
Biospecimen Consent	X	

Connect Fitbit	X	
Set Up Dexcom	X	
Administer Dexcom	X	
Upload Number for Mosio	X	
Administer ZIO® XT Patch	X	
Collect Saliva	X	
File Reimbursement		X

5.0.1 Participant Recruitment

Prospective candidates will be recruited through advertisements (i.e. IRB-approved posters and business cards), by word of mouth, or referral from medical or research staff. Potential study candidates will reach out to study staff by phone or email. Over the phone or email, study staff will confirm that the participant meets eligibility criteria before scheduling participants for a visit at Parnassus or Mission Bay. Informed consent will be obtained via Eureka prior to any study related procedures; the study participant will retain all consent documents through Eureka. Study participation is anticipated to be 14 days with two visits occurring at enrollment and week 2. The participant may withdraw voluntary participation at any time. An early termination visit should be done in the event that a participant chooses to discontinue participation prior to study completion.

5.0.2 Eureka Mobile Application:

We will use the Eureka mHealth Research Platform (CHR #16-19397) to facilitate single subject (N-of-1) experiments designed to answer key participant-identified research questions about personalized caffeine use.

In the event that Eureka is unable to support the study, or if a participant does not want to download the application, we will utilize the Mosio software, randomize on RANDOM.org, and connect the fitness tracker to Health eHeart (CHR #12-09993).

5.0.3 ZIO® XT Patch Event Monitor:

Each participant will be equipped with a ZIO® XT Patch, which is a 2" by 3" sticky patch. The event monitor utilizes validated algorithms to automatically detect heart rhythm disturbances independent of heart rate. The device records every heartbeat, providing a continuous Holter function. The device also includes a patient activator button, which we will ask participants to press to note coffee consumption. The ZIO® XT Patch is produced by iRhythm and mailed in a prepaid box back to iRhythm for analysis. iRhythm will not receive any patient identifying information. ZIO® XT Patch reports must be re-identified prior to uploading to the patient's electronic medical record.

5.0.4 Fitness Trackers: FitBit INSPIRE Tracker

Each participant will also be equipped with a fitness tracker, the FitBit INSPIRE tracker, to assess step counts and sleep. We will utilize an application programming interface (API) already developed and being used by the Health eHeart Study and Eureka platform to collect this fitness tracker data. As a part of the onboarding process, participants will also consent to share their fitness tracker data collected through their FitBit device, with our study through Eureka. In the event that participant does not want to download the Eureka application, we will connect the FitBit to Health eHeart (CHR #12-09993). Participants will consent to enroll in Module 5 "Linkage to health data from sensors to web applications".

5.0.5 Dexcom G6

In order to better understand how caffeine consumption fluctuations influence glucose levels, participants will also be given a continuous glucose monitor, Dexcom G6 CGM, to wear. These monitors are FDA approved. They are painless and adhere to the skin; CGM users insert a tiny sensor wire just under their skin using an automatic applicator. An adhesive patch holds the CGM sensor housing in place so the sensor can measure glucose readings throughout the day and night. The monitor measures glucose information in the interstitial fluid, which is a thin layer of fluid surrounding cells right below the skin, and collects glucose information every five minutes. In the event that the readings of the CGM go outside of the expected blood glucose range for nondiabetic individuals for a sustained period of time, a commercially available, FDA approved glucometer will be utilized to calibrate the CGM. In this process, a small finger prick is performed; a drop of blood is then placed on a test strip which is then inserted into the glucometer to take a reading. The value generated by the glucometer is then used to give feedback to the CGM.

5.0.6 Biospecimen Collection:

All patients will also have DNA collected via either saliva samples or blood work. They will undergo genotyping for single nucleotide polymorphisms previously shown to affect caffeine metabolism. Upon participant request, we will provide the results collected from the genotyping. However, we will inform participants that we will only be sharing information regarding caffeine metabolism that bears no clinical significance and only represents results from this research study (as opposed to clinical care).

Participants will consent to share their genetic information with our study through Health eHeart (CHR #12-09993). Participants will consent to enroll in Module 3 "Biospecimen Collection".

5.0.7 Geofencing Feature:

Participants will also consent to download and enable a feature on Eureka that allows investigators to identify the geolocation of participants' phones. This feature will collect geolocation data as long as location services are enabled on a participant's phone. Using a combination of GPS cell phone tower triangulation and local WiFi, the participant will be flagged as present in a publicly known location, i.e. a coffee shop, if they are within the vicinity of a location (such as within a distance to connect to that coffee shop's WiFi) for a certain period of time. This will allow for a novel and powerful assessment of how behavior changes when one is randomly assigned to consume or avoid caffeine. While our primary analysis will focus on visiting coffee shops (to validate our randomization assignment), we will also a priori geofence gymnasiums given evidence that individuals may exercise more when they consume caffeinated substances. Including geolocations of every type of publicly known location visited for at least 3 minutes though will allow for post-hoc and hypothesis-free analyses to enable discovery of clinically relevant patterns of behavior that may influence our outcomes of cardiac ectopy, exercise, and a subjective sense of well-being. This data will be deidentified and will only be collected for the duration of the study period.

5.0.8. Mosio Text Messaging Service

Mosio is a text messaging software for clinical research, that will be able to deliver daily text reminders with surveys to the participants. Participants will receive a text welcoming them to the CRAVE study upon enrollment, and daily text with surveys on coffee consumption throughout the monitoring period.

5.1 Enrollment Visit

The Enrollment Visit takes place on Day 0; participants are still free to consume caffeine per their typical habits the day that they enroll. At the Enrollment Visit participants will consent for CRAVE. Following consent, participants will download the Geofencing feature, consent for Biospecimen, and connect their FitBit INSPIRE. After all the consents are signed, the study coordinator will collect saliva.

After obtaining informed consent, study staff will conduct an initial interview asking patients about their demographics, medical history, and participants will complete a survey regarding

their baseline coffee intake, caffeine withdrawal symptoms, history of palpitations. They will also be asked what time of day they would like to receive texts with instructions for the next day and questions regarding their coffee consumption during the previous day.

Should interested subjects beyond the Bay Area and unable to travel to UCSF initiate contact with study coordinators and decide to participate in the study, the study coordinators will conduct the initial visit and follow up visit remotely, over the phone. All study related documents requiring signatures, including the informed consent form, HIPAA form, and reimbursement forms will be signed electronically via DocuSign, a secure digital platform that allows for the electronic exchange of documents. Participants will also be able to retain a copy of their signed documents electronically on DocuSign.

After the initial interview, the patient will download the Eureka application and will have a ZIO® XT Patch placed at UCSF Medical Center, or will be mailed and provided instructions to place the ZIO® XT Patch should the participant be unable to travel to UCSF. This version of the app and study will largely leverage the programming already completed for the I-STOP-AFib study (IRB#16-21154). Alternatively, we will utilize the Mosio software for participants who do not want to download Eureka. Participants will be provided with Tegaderms to help ensure the ZIO® XT Patch remains on for the full two weeks.

Patients will also consent for and download the geofencing feature, programmed to detect >3 minutes in the vicinity of any public location. Three minutes was selected so as to avoid geofencing every publically known location passed while, for example, driving or walking. All participants will be asked to save copies of their receipts and will be reimbursed for coffee products on all receipts with legible date and time stamps.

5.2 Monitoring Period

The monitoring period is Day 1-14. Participants will be receiving daily instructions at 08:00 and reminders at 20:00, as well as their daily surveys. The AM message includes instructions for the day, and daily surveys for caffeine use and sleep. The PM message includes instructions for the next day, and daily surveys for mood and headaches.

After Day 14, participants are welcome to resume their normal caffeine habits, and will be prompted to do so by the PM message. Participants will receive their final message on Day 15, to finish their last set of surveys and will be reminded to resume their normal caffeine habits.

5.3 Termination Visit

The Termination Visit can be scheduled any day after Day 14. At the Termination Visit, coordinators will collect back the ZIO® XT Patch and drop it off in USPS mail. Study compliance reviews and reimbursement for coffee expenses will also be conducted during this visit.

5.4 Early Discontinuation

If a participant wishes to discontinue participation, coordinators will collect the study devices and conduct an early termination visit if the participant is agreeable. Participants will be removed as a contact from the Mosio Text Messaging service and stop receiving surveys.

6.0 Risks and Benefits of the Study Procedure

This is a minimal risk study, and the risks are such that we think a participant can readily understand the risks and weigh the risks and benefits appropriately.

6.1 Potential risks:

ZioPatch uses adhesive to stick to the skin. There is a risk of skin irritation resulting from the adhesive. Continuous glucose monitors, including the Dexcom G6, work by inserting a tiny sensor wire just under the skin, and use adhesive to stick to the skin. Participants may feel discomfort from the initial wire insertion, and there is a risk of skin irritation resulting from the adhesive. Participants may experience minor discomfort during the fingerprick for the glucometer usage in calibrating the CGM. Participants may experience minor discomfort during venipuncture for biospecimen collection.

The study on the whole involves the risk of loss of privacy. Should an individual be linked specifically to a given disease or other characteristic associated with social stigma or other adverse consequences, there is an associated risk of embarrassment or social discomfort.

> The risk of loss of privacy in our study hosted by the Eureka Research Platform will be present for all persons participating. Loss of privacy could occur by compromise of the Eureka technical system, or if Eureka is required by law to disclose data to authorities, e.g. to prevent serious harm to the participant or others. This is the primary reason for the Eureka Privacy Policy and Data Security Measures.
>
> The risk of loss of privacy in our study associated with using Mosio will be present for all persons participating. Loss of privacy could occur by compromise of the Mosio technical system, or if Mosio is required by law to disclose data to authorities, e.g. to prevent serious harm to the participant or others. This is detailed in the Mosio Privacy Policy.

6.2 Potential benefits:

Health and lifestyle changes may occur as a result of participation. Participants may gain knowledge about their health and health conditions. Participants may have a feeling of contribution to knowledge in the health or social sciences field.

7.0 Study Roles

The CRAVE study has been developed by the Principal Investigator at the University of California, San Francisco. The study coordinators are committed to conducting this study in a uniform manner, adhering to the study protocol and the operations manual. Standardization, supervision and coordination of all procedures will be enhanced through peer review and quality control mechanisms.

Study visits and activities will occur at the UCSF Mission Bay and Parnassus Cardiology Clinics. We will also utilize the Clinical Research Center located at the Parnassus Campus to conduct patient visits.

8.0 Protocol Deviations

The Principal Investigator is required to adhere to the study protocol, applicable federal (national) or state/local, laws and regulations, and any conditions required by the IRB or applicable regulatory authorities.

Protocol deviation is used to describe situations in which the clinical protocol was not followed. All major deviations from the study protocol must be reported to the local IRB as appropriate, per the IRB's reporting requirements.

9.0 Quality Insurance and Data Management

9.1 Clinical Site Investigator and Coordinator Training

Each coordinator will be trained on the study protocol and procedures to ensure accurate and consistent study methods are used study-wide and throughout the entire study duration. Training will include review of the protocol, operations manual, and data management procedures.

9.2 Data Handling and Confidentiality

Information about study participants will be kept confidential and managed according to the requirements of the Health Insurance Portability and Accountability Act of 1996 (HIPAA). Those regulations require a signed patient authorization informing the patient of the following:

- What protected health information (PHI) will be collected from study participants
- Who will have access to that information and why
- Who will use or disclose that information
- The rights of a research participant to revoke their authorization for use of their PHI

In the event that a participant revokes authorization to collect or use PHI, the Principal Investigator, by regulation, retains the ability to use all information collected prior to the revocation of participant authorization.

9.3 Data Collection and Management

Information will be transmitted and stored using state-of-the-art security systems similar to those that protect websites used by banks and electronic health record systems. Specifically, the Eureka Platform is hosted on Amazon Web Services (AWS), a cloud-based server system and computing services that are HIPAA compliant, and Eureka follows security guidelines of the U.S. Health Insurance Portability and Accountability Act of 1996 (HIPAA). Specifically, all research data are stored behind a secure firewall, guarded by intrusion detection software, and encrypted at rest and in transit in our Amazon Virtual Private Cloud. The network, including all the servers that will store our research data, is behind a secure firewall that does not allow unauthorized access to any research data server.

The Eureka Privacy Policy and Data Security Measures statement will inform participants of the risks of loss of privacy, including via technical compromise or legal requirements. We will also make participants aware that they are responsible for keeping their login credentials secure.

To minimize risk of loss of privacy, Mosio holds to the following policies, derived from Mosio's Privacy Policy: "Personal information is never shared outside Mosio without permission, except under conditions explained below. Inside Mosio, data is stored in security-controlled, HIPAA-compliant servers with limited access. Information may be stored and processed in the United States or any other country where Mosio, its subsidiaries, affiliates or agents are located.

Mosio may send personal information to other companies or people under any of the following circumstances:

- When they have consent to share the information
- If sharing information is necessary to provide a product or requested service (If information is shared with third parties we only provide the information they need to deliver the service. Also, such companies are prohibited from using information for any other purpose)
- To keep users up to date on the latest product announcements, software updates, special offers or other information we think users would like to hear about (unless users have opted out of these types of communications)
- If required to do so by law, to enforce the Terms of Use, or in urgent circumstances, to protect personal safety, the public or their websites

An audit trail will record all logins and any changes to study data, and all study data is housed on a secure server. All servers are protected from viruses using anti-virus software. This software automatically checks for virus signature file updates once an hour, and if necessary, directly updates itself. All antivirus software is monitored and network personnel notified in the event that the software stops functioning on a server.

The study database is backed up regularly to ensure that no data is lost. Our disaster recovery system also follows Standard Operating Procedures to maintain full security.

10.0 Statistical Methods

10.1 Sample Size and Randomization

The goal is to enroll at least 80 participants with full completion of all study measures. Recruitment of 80 patients will be both feasible in terms of budget and sufficient in size to detect differences in ectopy in response to caffeine. CHS data on PACs/hour and PVCs/hour were used in power calculations to determine the minimal detectable percentage increase (MDPI) in ectopy with 80% power. The MDPI with a sample size of 20 was 70%, compared to 30% with 80 subjects.

We will perform in-person recruitment, supported by Dr. David Rosenthal, post-doctoral fellow, and Dr. Marcus' CRCs. Given the N-of-1 strategy, the effect of caffeine on a single patient can be combined in a multilevel statistical model to increase study power.

10.2 Statistical Analysis Plan (SAP) and Outcome Assessment

For our primary outcome, we will analyze differences in PAC and PVC counts on days participants were randomized to consume coffee vs. avoid coffee. As secondary outcomes, we will compare differences in SVT and VT episodes, mean daily glucose levels, mean step count, and mean sleep duration, also for days randomized to consume coffee vs. avoid coffee.

10.2 Intention-to-Treat (ITT)

The main intention-to-treat (ITT) analysis set will include all randomized participants, whether or not they are compliant with the treatment allocation for this trial. Every attempt will be made to collect data until the end of the follow-up period for all randomized participants and these data will be included as part of the main ITT analysis. The analyses for the primary objective will be performed using the ITT dataset.

As-treated analyses utilizing counts of coffee consumed inferred by the patient activation button on the Zio patch will be employed to assess quantitative relationships.

Although substantial crossover is not anticipated, per protocol analyses will also be conducted if failure to comply with allocated treatment assignments occurs in more than 10% of cases or if there is a significantly different proportion of assignment adherence in one group compared to the other.

11.0 Ethical Considerations

This study is to be conducted according to US and international standards of Good Clinical Practice (FDA Title 21 part 812 and International Conference on Harmonization guidelines), applicable government regulations and Institutional research policies and procedures.

11.1 Institutional Review Board (IRB) and Ethics Committee (EC) Approval

This protocol and any amendments will be submitted to a properly constituted independent IRB for each clinical site, in agreement with local legal prescriptions, for formal approval of the study conduct. The decision of the IRB concerning the conduct of the study will be made in writing to the Principal Investigator and a copy of this decision will be provided to the funder before commencement of this study. The Principal Investigator should also provide a list of IRB members and their affiliate to the UCSF CC.

11.2 Informed Consent

The investigator must explain to each participant the nature of the study, its purpose, the procedures involved, the expected duration, the potential risks and benefits involved and any discomfort it may entail. Each participant must be informed that participation in the study is voluntary and that he/she may withdraw from the study at any time and that withdrawal of consent will not affect his/her subsequent medical treatment or relationship with the treating physician.

This informed consent should be given by means of a standard written statement, written in non-technical language. The subject should read and consider the statement before signing and dating it, and should be given a copy of the signed document. If the participant cannot read or sign the document, oral presentation may be made or signature given by the participant’s legally appointed representative, if witnessed by a person not involved in the study, mentioning that the participant could not read or sign the documents. No participant can enter the study before his/her informed consent has been obtained.

The informed consent form must be submitted by the investigator for IRB approval. An informed consent template will be provided to all of the clinical sites for their use.

11.3 Declaration of Helsinki

The investigator must conduct the trial in accordance with the Declaration of Helsinki.

12.0 Protocol Amendments

Any change or addition to this study protocol, which significantly affects the safety of participants, the scope of the investigation or the scientific quality of the study, will require a

written protocol amendment. The site must receive IRB approval for the amendment, prior to implementation.

Bibliography

1. Manolio TA, Furberg CD, Rautaharju PM, Siscovick D, Newman AB, Borhani NO, Gardin JM, Tabatznik B. Cardiac arrhythmias on 24-h ambulatory electrocardiography in older women and men: the Cardiovascular Health Study. Journal of the American College of Cardiology. 1994; 23(4):916–925

2. Simpson RJ, Cascio WE, Schreiner PJ, Crow RS, Rautaharju PM, Heiss G. Prevalence of premature ventricular contractions in a population of African American and white men and women: the Atherosclerosis Risk in Communities (ARIC) study. American Heart Journal. 2002; 143(3):535–540.

3. Haissaguerre M, Jais P, Shah DC, Takahashi A, Hocini M, Quiniou G, Garrigue S, Le Mouroux A, Le Metayer P, Clementy J. Spontaneous initiation of atrial fibrillation by ectopic beats originating in the pulmonary veins. New England Journal of Medicine. 1998; 339(10):659–666.

4. Dewland TA, Vittinghoff E, Mandyam MC, et al. Atrial ectopy as a predictor of incident atrial fibrillation: a cohort study. Annals of internal medicine. 2013;159(11):721-728.

5. Binici Z, Intzilakis T, Nielsen OW, Kober L, Sajadieh A. Excessive supraventricular ectopic activity and increased risk of atrial fibrillation and stroke. Circulation. 2010; 121(17):1904–1911.

6. Massing MW, Simpson RJ, Rautaharju PM, Schreiner PJ, Crow R, Heiss G. Usefulness of ventricular premature complexes to predict coronary heart disease events and mortality (from the Atherosclerosis Risk In Communities cohort). American Journal of Cardiology. 2006; 98(12):1609–1612.

7. Agarwal SK, Simpson RJ, Rautaharju P, Alonso A, Shahar E, Massing M, Saba S, Heiss G. Relation of ventricular premature complexes to heart failure (from the Atherosclerosis Risk In Communities [ARIC] Study). American Journal of Cardiology. 2012; 109(1):105–109.

8. DeBacker G, Jacobs D, Prineas R, Crow R, Vilandre J, Kennedy H, Blackburn H. Ventricular premature contractions: a randomized non-drug intervention trial in normal men. Circulation. 1979; 59(4):762–769.

9. Dixit S, Stein PK, Dewland TA, et al. Consumption of Caffeinated Products and Cardiac Ectopy. Journal of the American Heart Association. 2016;5(1).

10. Blomstrom-Lundqvist C, Scheinman MM, Aliot EM, et al. ACC/AHA/ESC guidelines for the management of patients with supraventricular arrhythmias--executive summary. a

report of the American college of cardiology/American heart association task force on practice guidelines and the European society of cardiology committee for practice guidelines (writing committee to develop guidelines for the management of patients with supraventricular arrhythmias) developed in collaboration with NASPE-Heart Rhythm Society. Journal of the American College of Cardiology. 2003;42(8):1493-1531.

11. Chugh SS, Blackshear JL, Shen WK, Hammill SC, Gersh BJ. Epidemiology and natural history of atrial fibrillation: clinical implications. Journal of the American College of Cardiology. 2001;37(2):371-378.

12. Coyne KS, Paramore C, Grandy S, Mercader M, Reynolds M, Zimetbaum P. Assessing the direct costs of treating non valvular atrial fibrillation in the United States. Value in health : the journal of the International Society for Pharmacoeconomics and Outcomes Research. 2006;9(5):348-356.

13. Roger VL, Go AS, Lloyd-Jones DM, et al. Heart disease and stroke statistics--2012 update: a report from the American Heart Association. Circulation. 2012;125(1):e2-e220.

14. Dukes JW, Dewland TA, Vittinghoff E, et al. Ventricular Ectopy as a Predictor of Failure and Death. Journal of the American College of Cardiology. 2015;66(2):101-109.

15. Medscape. Ventricular Premature Complexes Treatment & Management. . 2014; http://emedicine.medscape.com/article/158939-treatment - a1130.

16. UpToDate. Ventricular Premature Beats. 2017; http://www.uptodate.com/contents/ventricularpremature-beats.

Summary of Protocol Changes

As an investigator-initiated study (not designed as part of a formal product investigation, such as for the Food and Drug Administration), the initial protocol was drafted as an operations manual. Subsequently, but prior to the final analyses and drafting of the manuscript, a protocol that more completely incorporated details included in the approved Institutional Review Board application and ClinicalTrials.gov was completed. Therefore, while the content of the written document changed, the study design itself did not.
