# Supplementary material for: Acute effects of caffeine withdrawal on headache among regular caffeinated coffee drinkers
Source: Sci Rep. 2026 May 22;16:23299. doi: 10.1038/s41598-026-54049-3 (PMC13402579; doi:10.1038/s41598-026-54049-3)
Supplement: Supplementary file 2 — Supplementary Material 2 [file 41598_2026_54049_MOESM2_ESM.docx]

**Supplemental Tables**

**Supplementary Table S1. Participants Completing Less than 14 Day Study Duration and Reasons for Early Withdrawal.**

| **Days of Study Participation** | **Number of Participants** |
| --- | --- |
| 3 | 1 |
| 4 | 1 |
| 5 | 3 |
| 9 | 1 |
| 10 | 2 |
| 11 | 3 |
| 12 | 5 |
| 13 | 3 |
| **Total: 19** | |

**Reasons for early termination were all due to premature removal of the continuous electrocardiographic monitor:**

- Skin irritation (n=5)
- Weakened adhesive (n=2)
- Unknown (n=12)

**Supplementary Table S2. Caffeinated** **Coffee Consumption vs. Caffeine Avoidance Day**

| **Comparison** | | **Frequency of Headache Occurrence** | **Risk Reduction (RR)** | **Headache Severity (RR)** |
| --- | --- | --- | --- | --- |
| Intention-to-treat | Coffee consumption | 10% (p<0.0001) | 0.34 (95% CI, 0.24-0.47; p<0.001) | 0.27 (95% CI, 0.22-0.33; p<0.001) |
|  | Caffeine avoidance | 30% (p<0.0001) |  |  |
| As-treated | Coffee consumption | 11% (p<0.0001) | 0.60 (95% CI, 0.49-0.73; p<0.0001) | 0.92 (95% CI, 0.78-1.1; p=0.37) |
|  | Caffeine avoidance | 31% (p<0.0001) |  |  |

**Supplementary Table S3. Coffee Avoidance Day Preceded by Coffee Consumption vs. Avoidance Day**

| **Comparison** | | **Frequency of Headache Occurrence** | **Risk Reduction (RR)** | **Headache Severity (RR)** |
| --- | --- | --- | --- | --- |
| Intention-to-treat | Coffee avoidance day preceded by coffee consumption day | 28% (p=0.31) | 0.84 (95% CI, 0.61-1.16; p=0.30) | 0.82 (95% CI, 0.58 -1.2; p=0.27) |
|  | Caffeine avoidance day preceded by caffeine avoidance day | 33% (p=0.31) |  |  |
| As-treated | Caffeine avoidance day preceded by coffee consumption day | 32% (p=0.59) | - | 1.03 (95% CI, 0.81-1.3; p= 0.79) |
|  | Caffeine avoidance day preceded by caffeine avoidance day | 29% (p=0.59) |  |  |

**Supplementary Table S4. Caffeine Avoidance Day Preceded by One vs. Two Days of Caffeinated Coffee Consumption**

| **Comparison** | | **Frequency of Headache Occurrence** | **Risk Reduction (RR)** | **Headache Severity (RR)** |
| --- | --- | --- | --- | --- |
| Intention-to-treat | Caffeine avoidance day preceded by one coffee consumption day | 32% (p<0.0001) | 0.57 (95% CI, 0.37-0.88; p=0.01) | 0.59 (95% CI, 0.43-0.81; p<0.001) |
|  | Caffeine avoidance day preceded by two coffee consumption days | 18% (p<0.0001) |  |  |
| As-treated | Caffeine avoidance day preceded by one coffee consumption day | 35% (p<0.0001) | - | 0.71 (95% CI, 0.47-1.1; p=0.092) |
|  | Caffeine avoidance day preceded by two coffee consumption days | 23% (p<0.0001) |  |  |

**Supplementary Table S5. Association between caffeinated coffee consumption and evening headache occurrence within each tertile of caffeine metabolizer polygenic score.**

| **Polygenic score tertile** | **Odds ratio** | **95% CI** | **P-value** | **Interaction p-value** |
| --- | --- | --- | --- | --- |
| Slow | 0.11 | 0.04 to 0.32 | <0.0001 | 0.34 |
| Intermediate | 0.20 | 0.10 to 0.38 | <0.0001 |  |
| Fast | 0.24 | 0.14 to 0.43 | <0.0001 |  |

Odds of headache in the presence (versus absence) of caffeinated coffee.

**Supplementary Table S6. Association between caffeinated coffee consumption and evening headache occurrence within stratum of baseline headache status.**

| **Baseline Headache Status** | **Odds ratio** | **95% CI** | **P-value** | **Interaction p-value** |
| --- | --- | --- | --- | --- |
| No baseline headache | 0.29 | 0.17 to 0.51 | <0.0001 | 0.043 |
| Baseline headache | 0.13 | 0.08 to 0.23 | <0.0001 |  |

Odds of headache in the presence (versus absence) of caffeinated coffee.

**Supplementary Table S7. Association between caffeinated coffee consumption and evening headache occurrence within each stratum of baseline caffeinated coffee consumption.**

| **Baseline caffeinated coffee consumption** | **Odds ratio** | **95% CI** | **P-value** | **Interaction p-value** |
| --- | --- | --- | --- | --- |
| Occasional | 0.63 | 0.14 to 2.78 | 0.54 | 0.001 |
| Regular | 0.289 | 0.29 to 0.29 | <0.0001 |  |
| Heavy | 0.043 | 0.016 to 0.11 | <0.0001 |  |

Odds of headache in the presence (versus absence) of caffeinated coffee.
